# Knowledge and Practices of Cypriot Bovine Farmers towards Effective and Safe Manure Management

**DOI:** 10.3390/vetsci10040293

**Published:** 2023-04-14

**Authors:** Soteris Christophe, Kristina Pentieva, George Botsaris

**Affiliations:** 1 Cyprus Veterinary Services, Athalassas Av., Nicosia 1417, Cyprus; 2 School of Biomedical Sciences, Ulster University, Coleraine BT52 1SA, UK; 3 Department of Agricultural Sciences, Biotechnology and Food Science, Cyprus University of Technology, Limassol 3603, Cyprus

**Keywords:** bovine farmers, manure management, knowledge, practices, manure treatment

## Abstract

**Simple Summary:**

Manure generated in bovine farms may pose human and animal health risks. If not managed and used properly, these risks may be spread to other areas. The risks can be minimised when farmers are aware of them and apply optimum practices at their farms, at storage, processing, and when manure is applied to land as a fertiliser. This research aims to evaluate the Cypriot bovine farmers’ knowledge about the risks from manure and the practices currently applied for manure management through a questionnaire survey. The results indicated some gaps in farmers’ knowledge and some deficiencies in employing optimal management practices. The education level and the farming purpose were identified as determinants of the level of farmers’ knowledge. The conclusion was that farmers’ knowledge must be reinforced through specialised training to ensure proper manure management. Although the current practices partially decrease manure pathogens, interventions to promote the use of more effective treatment methods, such as biogas transformation and composting, would be beneficial. The results could help Cypriot and other competent authorities to develop an action plan to control the health risks by educating farmers, promoting the use of more efficient management methods and developing new legislation.

**Abstract:**

Manure from bovine farms is commonly used as an organic fertiliser. However, if not properly managed, it can spread significant biological and chemical hazards, threatening both human and animal health. The effectiveness of risk control hugely relies on farmers’ knowledge regarding safe manure management and on the application of suitable management practices. This study aims to evaluate the knowledge and practices of Cypriot bovine farmers towards safer manure management, from its generation to its final use, in line with the One Health approach. Factors affecting farmers’ knowledge and applied practices are also investigated through a questionnaire survey. The questionnaire was developed and sent to all eligible bovine farmers in Cyprus (n = 353), and 30% (n = 105) of them returned the completed questionnaire. Results revealed there are some gaps in farmers’ knowledge. The use of manure for fertilising crops dominated. Only half of the farmers stored manure in appropriate facilities, with 28.5% of them using a dedicated area with cement floors and 21.5% utilising leakproof tanks. The majority (65.7%) stored manure for more than three months before its use as a fertiliser in a dried form. In multiple regression analysis, education level and farming purpose were significant determinants of farmer knowledge. In conclusion, Cypriot farmers’ knowledge must be reinforced to ensure proper manure management. The results highlight the importance of providing relevant training to farmers. Although the current practices partially decrease manure pathogens, interventions to promote the use of more effective treatment methods, such as biogas transformation and composting, would be beneficial.

## 1. Introduction

Manure is among the most hazardous livestock farming by-products for human and animal health as it contains a substantial amount of pathogens [1] that present severe biological and chemical hazards [2]. In the EU and UK together, 1.4 billion tonnes of manure are generated each year, of which 75% is of bovine origin [3]. Because of the limited storage and processing facilities in farms, about 92% of the manure is applied directly to land unprocessed [3]. Cyprus is facing similar problems—in its five districts, the bovine population in 2021 was approximately 84,000 animals, and it is estimated that they generated about 1,125,000 tonnes of manure, representing more than half of total manure quantities in Cyprus [3]. There are no official data for the final disposal or use of this manure, but there is evidence that the most common practice is the storage in deep pits and anaerobic lagoons before land application [4].

Manure may harbour a variety of pathogens; bacteria, including *Salmonella* spp., *Campylobacter* spp. and *E. coli*; viruses, including apthoviruses, enteric viruses, and myxoviruses; and various parasites, mycoses and yeasts [5,6,7,8,9,10]. It has been implicated in several incidences of spreading diseases such as *E. coli* (0157:H7) diarrhoea, Cryptosporidiosis, and Salmonellosis [11,12,13]. Additionally, chemical substances that are routinely used or are unintentionally present in farming may be traced in manure. Consequently, it may contain antibiotic residues [14,15,16,17,18] and antibiotic-resistant bacteria [19,20,21] or other contaminants, including dioxins [22], heavy metals [23], illegal drugs [24] and hormones [25,26,27,28,29]. Improper manure storage, handling, and management may pollute the environment and expose humans and animals to the aforementioned hazards [9] via contaminated food, water, dust, wild animals, pests and others [5,10,30,31]. Traditionally, manure was used for fertilising crops and improving soil quality [9,32]. The intensification of breeding systems has led to excess manure production, which is above the local needs. This necessitates the storage of manure on the farm and the dispatch to other regions for application [33], which in turn, promotes the spreading of pathogens [9].

Risks to human and animal health can be mitigated by applying good management practices and taking prevention measures in three stages [10]. The first is the application of good farming practices to reduce the initial manure’s pathogen load and hazardous residues. These include pest control programmes, good husbandry practices, maintaining an ideal level of animal welfare, the prudent use of veterinary medicines, a vaccination programme, parasite control and others [34,35,36]. The second level is based on chemical, physical, or biological manure decontamination methods. These reduce health risks [9] despite being developed for other purposes such as volume reduction and profitability [37]. Among manure decontamination methods, the pasteurisation at 70 °C for 1 h following biogas transformation or aerobic composting is distinguished for its effectiveness [32,37,38,39,40]. Finally, it is important to follow the best practices during manure’s use as a fertiliser, including prompt incorporation into land and avoiding sloping grounds to prevent run-off after intense precipitation [10,41,42,43,44,45]. The effect of the sun’s UV radiation, the soil bacteria and desiccation [10,46] play a key role in reducing pathogens.

Historically, manure is used directly on land or after a certain storage period. Consequently, there is minimal pathogen reduction, if any. The European Parliament and European Council Regulation (EC) No. 1069/2009 and Commission Regulation (EU) No. 142/2011, commonly referred to as the animal by-products regulations, allow for the direct application of manure to land without any prior treatment within the territory of the member state if the competent authority approves such use. Therefore, the application of unprocessed manure on land is a common practice among EU member states. Although Regulation (EU) 2019/1009 allows the use of animal by-products, including manure in fertilising products provided that are to be treated to a specified level of safety defined in the animal by-products regulations, it does not exclude the use of unprocessed manure within the member state of production if not used to produce fertilising products. The national legislation of Cyprus for manure management focuses on environmental issues. Specifically, it consists of two acts issued based on the Law for the Control of Water Pollution 106(I)/2002, namely the Regulatory-Administrative Act 263/2007 for “Good Agricultural Practices” and the Act 433/2006 for the General Rules for the Disposal of Waste from Bovine Farms” aiming against the nitrate pollution of underground waters. The acts set rules for storing and managing manure in the farm and its use on land as a fertiliser. Among others, they recommend three-month storage of the manure before application on land, cement floors for storage of manure in the farm with a slope towards a leakproof tank for separation of the liquid fraction and quicker air drying of the manure. Consequently, the risk of spreading live pathogens and other hazardous agents from the use of manure is expected to remain a significant challenge.

While health risks arising from the improper use or disposal of manure and incidences of zoonotic outbreaks related to contaminated manure are being studied intensively [7,9,10], the safe management of manure remains a significant problem [47,48] since data about farmers’ knowledge and applied practices for safe manure management are scarce. To the best of our knowledge, so far, there is no research investigating the knowledge of farmers regarding human and animal health risks associated with improper manure management and current management practices in Cyprus and other European countries, which is a serious gap in this field. Considering the significant contribution of bovine manure the total manure production, this study aims to evaluate bovine farmers’ knowledge about public and animal health risks associated with manure generated in their farms and to identify current practices applied for manure management in Cyprus. Furthermore, it aims to investigate the demographic and other determinants that affect the farmers’ knowledge and applied practices, so appropriate interventions can be implemented to improve the safety of manure management following a holistic approach to the issue, starting from breeding practices to the final use, contributing towards the One Health perspective.

## 2. Materials and Methods

### 2.1. Study Population

To evaluate the knowledge and practices of bovine farmers towards effective and safe manure management, a questionnaire survey was conducted. The study population included all farmers of bovine animals who have active animal holdings in the areas under the effective control of the Government of the Republic of Cyprus. The necessary information, including farmers’ contact details, was obtained from the database of the Veterinary Services of Cyprus. Bovines were kept in 384 farms (average farm size: 220 animals), belonging to 374 physical or legal persons [49]. In 2021, 21 farms were focusing on beef production exclusively, whereas all the rest were dairy farms or dairy mixed with beef. Dairy breeds, mainly Holstein-Friesians, represent more than 97% of the population. A small number of animals belong to Jersey, Limousin, Black Angus, the local red breed, Swiss Brown, Charolais and other breeds. The animals are kept confined in farms except for about 200 animals belonging to the local breed in the pastureland of Akrotiri of Lemesos District [50]. From the 374 farmers listed in the database, 21 organisations, research institutes and farmers currently with no animals were excluded. All other farmers in the database, 353, were eligible for participation and invited to contribute to the research.

### 2.2. Questionnaire Development and Administration

The questionnaire was developed and designed de novo to evaluate the knowledge and practices followed during the three critical stages for controlling the risks from manure [51]; in particular, to evaluate the application of good farming practices, the use of decontamination methods before manure use, and the practices followed when it is applied to land. It comprised three sections: Demographics (farmer’s age, years in the profession, education and farm location, size and farming purpose), Assessment of Knowledge, and Identification of Current Practices/Approaches (Appendix A). The questionnaire was written in Greek, which is the language spoken in Cyprus. It included a combination of question types; for example, dichotomous questions (yes/no; right/wrong); Likert scale questions (“Not at all important”, “Slightly important”, “Important” and “very important”); and free numerical responses (for example “Please state your age”). For the assessment of the knowledge, a scoring system was developed analogous to that of Tierney et al. [52], who allocated a point for each correct answer. That part of the questionnaire consisted of 4 main questions, each having several sub-questions, for a total of 31 questions. The questions were related to safe manure management practices, diseases and other hazards transmitted through manure, treatment methods to reduce the risks, and general right or wrong statements. The highest score a responder could get was 31 points (6 points for question 1, 11 for question 2, 8 for question 3 and 6 for question 4).

The questionnaire was piloted on ten independent persons from diverse backgrounds but relevant to the field to check it for clarity, simplicity and precision [53]. Based on their feedback, the questionnaire was revised accordingly and finalised. The estimated completion time was five to ten minutes. After the ethical approval was granted, a package containing an invitation letter providing information about the study, together with the questionnaire and a stamped (prepaid), self-addressed envelope for returning the completed questionnaire to the researcher, was sent to farmers. The invitation letter explained the study’s scope, participants’ rights and confidentiality issues, researcher’s contact details, instructions on how to return the completed questionnaire and other relevant details. To ensure the anonymity of the survey, the questionnaire did not include any questions or other elements that could lead to the identification of responders. The questionnaire distribution started in May 2022, and responses were received until mid-July 2022. Some of the envelopes were distributed by a farmers’ organisation to its members and some by personnel of the Veterinary Services while visiting farms for their routine work. Care was taken so that those farmers were not approached for the same survey by the researcher. No other attempt was made to contact participants, and no compensation of any kind was offered to responders. The initial telephone approach was excluded to protect the personal data of the target group, despite the positive effect on the response rate [51,54].

### 2.3. Statistical Analysis

Data were analysed using IBM SPSS statistics for Windows, version 27 [55]. Descriptive statistics were performed for demographic characteristics and to describe responses concerning knowledge. All records were examined for being within the expected values, for missing values and for any other errors. Outliers were identified, verified that they were not attributed to errors and kept for statistical analysis. Differences between participants’ categories and between applied practices for safe manure management were assessed using the Chi-Square test.

The distribution of the “Total knowledge score” was checked for normality using the Kolmogorov–Smirnov and the Shapiro–Wilk tests. The tests indicated that there was a deviation from normality (*p* < 0.001). It was not possible to normalise the distribution with data transformation. As a consequence, to compare differences in knowledge scores between demographic groups, the Mann–Whitney U Test was used as an alternative to the Independent Samples *t*-test. The farm size, education level, experience, and farmers’ age were dichotomised for the analysis. Farm size was divided into farms with equal or less than 250 bovines and those with more than 250 animals because the bigger farms, typically, are managed by professional farmers, whereas smaller farms are managed by farmers who practice farming as a secondary occupation. The education level, farmers’ age, and experience, which are reflected in the years in the occupation, were divided at the median value. The Kruskal–Wallis Test was used to compare the variance of the total score between the districts. To further investigate whether various demographic characteristics are determinants of participants’ knowledge scores, multiple linear regression analyses were performed with the knowledge score as the dependent variable and the farming experience, education level, herd size and farming purpose as independent variables. Additionally, the variable of “Farming Purposes” was used after merging the “dairy purpose” with “a mixed production purpose” (dairy and beef) because all mixed-purpose farms in Cyprus have cattle that belong to dairy breeds, and they are raised as an appendant activity.

To compare the knowledge of groups with different beliefs and attitudes towards good farming practices, the Mann–Whitney U-Test was used. To perform the test, Likert scale questions were dichotomised by merging “Not at all Important” with “Slightly Important” and “Important” with “Very Important” answers [56,57]. The “Not sure/No opinion/Don’t know” answers were treated as blanks.

For all analyses, the *a*-value was determined at 0.05, and any *p*-value less than 0.05 was considered significant. Missing data were considered blanks and were excluded pairwise from analyses.

## 3. Results

### 3.1. Description of the Responders

Of the 353 farmers approached, 105 returned completed questionnaires, representing a response rate of 30%. The general characteristics of the participants are presented in Table 1. Most of the participants were in the age group of 55–64 years. Most of the farmers had long farming experience, usually more than 30 years and had finished secondary education (Lyceum). Responders were predominantly from the Lefkosia district, following Paphos and Larnaka. The majority had a mixed farming purpose (dairy and beef). Farms having up to 250 animals predominated.

### 3.2. Description of Practices for Manure Management

About 85% of participants used manure as a fertiliser. Almost 70% of those using it are in dry form. Nearly one-third of stored manure is in piles at a dedicated area of the farm, usually with cement floors. The majority of participants stored manure for more than three months before dispatching it to be used as a fertiliser without any further treatment (Table 2).

### 3.3. Evaluation of Knowledge

The mean knowledge score achieved by participants at the four types of questions as well as the total score, are presented in Table 3. For most questions, the score was above 50% of the maximum, apart from the question concerning the transmission of diseases and other hazards from contaminated manure, which was 43.5% of the maximum score. The score did not approach full marks in any of the questions, with the highest achieved being 68.5% of the maximum in the question about good farming practices. A detailed breakthrough of the answers to each of the questions is presented in Appendix B (Table A1).

### 3.4. Participant Characteristics in Relation to Knowledge Level and Applied Practices

Comparing the median scores of farmers with different characteristics revealed that participants managing larger farms, participants managing dairy or dairy with beef cattle farms, and participants with an educational level of lyceum and above had significantly higher scores. Farmers from the Paphos district scored significantly lower than other districts. A detailed comparison of scores obtained by farmers with different characteristics is presented in Appendix B (Table A2). Further analysis to investigate whether the farming experience, education level, herd size and farming purpose were determinants of the knowledge score was carried out. The results of the multiple linear regression analysis suggested that the education level and the farming purpose could significantly influence the score (Table 4). In particular, the higher the education level, the higher was the score. Likewise, farmers who kept only animals of the local breed had lower scores than farmers who kept animals for beef purposes only, and the latter group scored lower than those who kept dairy animals or animals for mixed, dairy and beef purposes. The age was excluded from the analysis because of significant collinearity with farming experience. The district of the farm was also excluded because of the presence of outliers. The final model, which included only the two significant predictor variables, indicated that it could explain 39.3% of the variance of knowledge score [F(2, 92) = 31.46, *p* < 0.0005, R^2^ = 0.39]. Both variables, education level (B = 1.66, *p* = 0.03) and farming purpose (B = −4.287, *p* < 0.0005), contributed significantly to the model.

Farmers who had positive attitudes and beliefs towards specific good farming and manure management practices had a higher knowledge level about proper manure management (Table 5). In particular, farmers who considered vaccination, mice control programmes, animal welfare and proper manure management important for farming achieved significantly higher scores than those who did not consider these issues important. Regarding treatment methods, those who did not identify certain manure methods that increase safety as important achieved significantly lower scores. Specifically, the groups which considered not important the storage in piles (with mixing to promote fermentation), the storage in lagoons or tanks for at least six months, composting and air-drying under the sun had significantly lower scores than other groups. The scores of groups with different beliefs about pasteurisation, transformation into biogas, and addition of chemicals as methods for reducing manure risks did not show any significant differences.

## 4. Discussion

The results of the study characterise the level of knowledge about safe manure management and the practices used for that purpose by bovine farmers in Cyprus. The questionnaire analysis showed that despite the certain level of knowledge among farmers about safe manure management, they underestimate the importance of manure in spreading common pathogens, and more than half do not realise manure’s role in spreading serious infectious diseases. The results indicated that the farming purpose and the educational level of farmers are associated with better knowledge of the risks arising from improper manure management, whereas farmers’ age and total years in the occupation do not play a significant role. There are also geographical areas where the level of knowledge is lower than others. Regarding current practices, the use of manure as a fertiliser for crops is prominent. Typically, bovine farmers store their manure before dispatch. The fact that the manure is stored uncovered in a dedicated area with a cement floor, where it remains for more than three months until it becomes dehydrated, diminishes its pathogen levels.

Farmers’ knowledge about health risks from manure is extremely important to avoid practices that may spread diseases [59]. It appears that most farmers have a certain degree of knowledge about the risks arising from improper manure management. Šūmane et al. [60] indicated the importance for livestock farmers of having a certain knowledge level on new developments to maintain sustainability and resilience. Although there is no benchmark to compare knowledge on manure management, it would be beneficial if farmers were more aware of possible risks and the relevant mitigation measures. Responses to the questionnaire revealed some misconceptions about farmers in relation to the risks. In particular, their knowledge about diseases spreading through manure is not adequate considering that they achieved, on average, less than half of the maximum overall score. Farmers underestimate the importance of manure in spreading common pathogens, such as those responsible for enteritis and intestinal parasites, but also more than half do not realise manure’s role in spreading serious zoonotic and other infectious diseases that might have devastating effects on the sector, such as foot and mouth disease. This agrees with the finding of Eltayb et al. [61], who demonstrated that only half of the farmers in their study had knowledge of zoonotic diseases. Although these diseases are considered exotic in Cyprus, appropriate biosecurity measures should be in place to prevent the occurrence or diminish the consequences of any outbreaks [62].

There are certain specific practices that may reduce manure’s pathogen loads [10]. The detailed analysis of the results showed that farmers had some difficulties in correctly identifying practices that promote the reduction of pathogen loads, namely vaccination programmes, waiting for manure to become digested before using it as a fertiliser or soil improver and maintaining a high level of animal welfare. Regarding knowledge about manure treatment methods, almost two-thirds of participants were aware that these contribute to the reduction of pathogens. The effectiveness of manure’s storage in lagoons or tanks for a period of time seems to be underestimated. Some confusion also exists on matters such as the timing for manure use in crops. Overall, farmers have some knowledge gaps about proper safety practices in all stages of production, from the farming practices to storage, treatment and use of manure. The above results align with the conclusions of Ström et al. [59], who also pointed out that the lack of knowledge is a key element hindering safe manure management. In general, the knowledge about proper manure management must be reinforced, giving emphasis to areas of weakness, such as the transmission of certain diseases and good farming practices.

Implemented practices for manure management are extremely important for safety [10]. Among participants, the application of certain practices for manure management is prominent. As a rule, manure produced in bovine farms is used on land as a fertiliser. Very few farmers submit it to composting or biogas transformation at an approved plant prior to its use. Because these treatments reduce pathogens, especially when equipped with a pasteurisation unit [63,64], the importance of applying other good management practices becomes obvious. Unfortunately, only a negligible number of farmers perceived the prospect of utilising manure as an energy source in biogas plants. This supports the findings of Meyer et al. [65], who reported only one out of 394 farms surveyed used biogas transformation before manure was used as a fertiliser. This might be attributed to insufficient information, absence or deficient capacity of biogas plants nearby [66,67]. Promoting biogas and composting plants in areas with farming activities provides a sustainable alternative to managing manure, which enhances safety while increasing profitability in an environmentally friendly manner [66,68]. Possible financial constraints, lack of guidance, state aid, or infrastructures, insufficient legislation or other reasons may explain why farmers have restricted access to those plants and justify their approach to manure management. Therefore, more research is needed to provide insights for better utilisation of these methods, including the investigation of the above factors and their influence on the development of the methods.

Current practices before manure use on land, though not optimal, contribute to the sanitisation of the material. The majority of farmers store manure on the farm before use. The storage is usually done in a dedicated area, often with cement floors or in watertight tanks, which prevent contamination of the environment. This practice deviates from previous studies [7,69] that place large lagoons as the most common system of storage. This could be attributed to the implementation of the national environmental rules, which determine the accepted methods for in-farm storage [70]. The storage period varies, but as a rule, it exceeds three months. Microbiological processes during the prolonged storage period enable the aging or maturation of manure and the reduction of pathogens. The dehydration occurring during the process eliminates the available water for bacteria proliferation [9,10]. In conclusion, applied practices, which are driven mostly by the national environmental legislation, reduce the risks from pathogens and other contaminants, although they do not ultimately ensure safety for the environment, human and animal health.

Participants with specific characteristics achieved higher scores on knowledge about safe manure management. These include the size of the farm, farming purpose, area of the farm and the participants’ educational level. Wang et al. [71] and Singh et al. [72] indicated the relevance of education to the level of knowledge for the use of pesticides in farming. The multiple regression analysis has shown that the farming purpose and the farmer’s educational level have a significant effect on knowledge scores. The fact that a farmer’s age and years of experience do not seem to play a significant role suggests that life and farming experiences are not so important for the knowledge level as the specialised training. This points out the necessity of providing training to all farmers, emphasizing the importance of safe manure management to human and animal health and the environment. These findings also agree with Singh et al. [72], who evaluated farmers’ knowledge of zoonotic diseases; however, they indicated that there was a negative association between the age of participants and knowledge. Hundal et al. [73] concluded that age, education level and farm size are not significant determinants of knowledge about zoonotic risks. However, Sumane et al. [60] supported that experiences and own experimentation should not be underestimated. Usually, the farm size is linked to the level of professionalism. Farmers with smaller farms might practice farming as a second occupation, whereas bigger farms are usually operated by professional farmers, and the farming system is more intensive. This is particularly true about very small farms, usually with less than six animals. Farmers who receive their sole income from farming may be more concerned about safety risks and try to educate themselves more. The farming purpose also correlates with professionalism. Professional farmers usually manage dairy farms. Often, they keep male calves for beef production. Therefore, they have a mixed farming purpose. In Cyprus, rearing cattle for beef production, as a sole activity, is not as profitable as dairy farming. Furthermore, only dairy breeds are available for beef production. Consequently, it is common for exclusively beef cattle farmers to practise in parallel to other occupations. The same applies to farmers of bovines belonging to the local breed, which is the reason that rearing takes place in villages by non-professional farmers. A lot of these farms are encountered in the Paphos district, where the majority of bovine farms have few animals. These might be reasons for the poor results of this district compared with all other districts. Saha et al. [74] indicated the negative influence of small herd size on the knowledge level of animal rearing practices; however, there are studies supporting that herd size is not affecting knowledge [73]. In addition, the absence of any bovine farmers’ association in the area may deprive farmers of the opportunity for organised training [60]. The limited number of demographic factors identified as contributing to the knowledge level and the percentage of variance explained by the regression models indicate that further research is needed to detect other factors affecting the knowledge and practices used, including sources of information available to farmers, whether they have previously received relevant training, the relevance of their studies and others.

In our study, most farmers who considered good farming practices and some manure treatment methods not important achieved significantly lower scores than those who believed that these good farming practices are important for reducing the risk arising from manure. Although it is not clear what reasons shaped these attitudes, insufficient knowledge could be one of them, as Musallam et al. [75] demonstrated. The absence of significant differences between groups with different beliefs about the treatment methods of pasteurization and transformation into biogas is noteworthy. These methods are considered among the most effective against pathogens of manure, especially in combination are very efficient for both biological and chemical hazards effectiveness [32,37,38,39,40]. The absence of differences in scores between the groups reveals that farmers with both high and low scores might have equally incorrect beliefs and unveils the confusion about these methods. This indicates the lack of relevant information to farmers and the gap of knowledge that exists about the best available technics for controlling the hazards found in manure before using it as a fertiliser. Therefore, educating those farmers might contribute to the change in beliefs and attitudes and promote the application of safer farming practices [76].

To reinforce the attenuation of enzootic diseases and prevent new diseases, the parties involved in farmers’ training need to inform farmers about the health and environmental risks associated with manure and the methods that can be applied to mitigate those risks. Competent authorities, private veterinarians and farmers’ organisations can contribute to this by organising training sessions, awareness campaigns, preparing educational material and informing farmers accordingly [9,65,77]. Equally important is farmers’ networking, exchange of experiences and developing of practical skills [60]. The outcome of this research indicates that training is needed for all farmers. It also identifies the areas where training should focus on, the farmers’ groups that would particularly benefit, and provides guidance for appropriate interventions. Furthermore, the regulatory authorities may consider the amendment of the legislative framework for encouraging the application of manure treatment methods, setting microbiological criteria before manure use on land, introducing subsidies to develop biogas and composting plants and providing advice for running those facilities.

The main strength of this research is that it is the first attempt to gather information about farmers’ knowledge of safe manure management. The exclusion of phone and personal contact with participants reduced the potential bias of study results. The tool of the survey, which was a printed questionnaire, was more user-friendly to people unfamiliar with modern technologies. Although the questionnaire was completely anonymous, some farmers might be reluctant to participate for fear of spotting them as having limited knowledge or not implementing proper practices for manure management. Therefore, the average knowledge score and the safety of applied practices could be overestimated. This might contribute to the dichotomised possible answers (right/wrong, yes/no). Although this facilitated the completion of the questionnaire, it could lead to bias because the responders had a 50% chance of selecting the correct choice, even if they did not really know the answer. In addition, the information that the questionnaire collected for the applied farmer practices regarding the storage of manure could have been more detailed. A potential limitation of the study is the lack of information on the precise number of animals on each farm, which restricted the ability to examine differences in subgroups of herd sizes, especially in the smaller herd sizes (up to 250 animals). A further limitation is that the time the survey was executed coincided with a busy period for farmers, which might deter them from participation. Nevertheless, the 30% response rate was higher than that of Benjamin et al. [57], who followed the same approach but achieved a 26% response rate. Other studies reported slightly higher response rates; however, they encourage participation by providing incentives [78] or by telephone contacts [51,54].

Because of the distinctive circumstances of Cyprus, such as the use of dairy breeds for beef production, climatic conditions, restricted access to manure processing facilities, local farmers’ organisation schemes and the restricted island area, the generalisation of the results should be done with caution to settings and locations with different circumstances. Nevertheless, this study could be useful as baseline information for further similar research at other locations.

## 5. Conclusions

Despite the certain level of knowledge among farmers about public and animal health risks associated with manure management, their knowledge should be reinforced. Educating farmers may improve current management practices, which are not optimal for ensuring the safe use of manure and the sustainability of the sector. By promoting safe manure management, the sector intensifies its sustainability and public acceptance because of the lower impact on the environment and human and animal health. Authorities and other stakeholders should intervene to improve knowledge and promote safe practices. Training and education of farmers as well as promoting appropriate manure treatment methods, should be the core elements of these interventions. The farming purpose and educational level, as the most important factors associated with a high knowledge level could guide the prioritisation of training interventions.

## Figures and Tables

**Table 1:** General characteristics of participants.

Variable	Category	n	%	*p*-Value ^1^
Age (years)	18–24	102	1	<0.001
	25–34		6.9	
	35–44		23.5	
	45–54		24.5	
	55–64		33.3	
	>65		10.8	
Farming experience (years)	<10	100	13	<0.001
	10–19		11	
	20–29		19	
	30–39		27	
	40–49		26	
	>50		4	
Education	Primary (Elementary)	105	6.7	<0.001
	Gymnasium		20	
	Lyceum		53.3	
	University		19	
	None of the above		1	
District of the farm	Lefkosia	103	30.1	<0.001
	Ammochostos		12.6	
	Larnaka		22.3	
	Lemesos		6.8	
	Paphos		28.2	
Herd size (number of animals)	1–6	105	6.7	<0.001
	7–250		58.1	
	251–500		30.5	
	501–750		1.9	
	751–1000		1.9	
	>1000		1	
Farming purpose	Dairy (exclusively)	104	15.4	<0.001
	Beef (exclusively)		2.9	
	Mixed		62.5	
	Local breed		19.2	

n—Number of total responses in each category; %—Percentage of the total in each category. ^1^ The *p*-value was obtained by analysing the data using the Chi-Square Goodness of Fit test.

**Table 2:** Practices applied by participants for manure management.

Question	n	Possible Answers	Number of Answers	%	*p*-Value ^1^
How do you use manure produced on your farm?	94	As a Waste	5	5.3	<0.001
		As a Fertiliser	80	85.1	
		As an Energy Source	1	1.1	
		Any other uses	8	8.5	
How often do you send manure from your farm for final use?	102	Every week	12	11.8	<0.001
		Every month	12	11.8	
		Every quarter	11	10.8	
		More than three months	67	65.7	
In case the manure is used as a fertiliser, in which form is it used?	101	Liquid form	4	4	<0.001
		Air dried form	70	69.3	
		Both liquid and air dried	27	26.7	
How do you store manure at your farm before you send it to another place?	130	In a watertight tank	28	21.5	<0.001
		In uncovered piles	32	24.6	
		In covered piles	6	4.6	
		In a dedicated area with cement floor	37	28.5	
		In a place with earthen ground	15	11.5	
		In any place that is convenient	5	3.8	
		I don’t store it at all	7	5.4	
To where do you dispatch the manure produced on your farm?	103	To be used directly as a fertiliser in the fields	88	85.4	<0.001
		To a biogas plant	3	2.9	
		To become fertiliser in a composting plant	10	9.7	
		To be disposed of as a waste	2	1.9	
		To be incinerated	0	0	

n—Number of total responses in each question; %—Percentage of the responders who chose each answer. ^1^ The *p*-value was obtained by analysing the data using Chi-Square Goodness of Fit test.

**Table 3:** Participants’ knowledge concerning safe manure management ^1^.

Question	Mean Score	SD ^2^	% of Maximum Score ^3^
Which actions do you believe help in reducing the risk of transmission of diseases to animals or humans from the improper manure management?	4.1	0.87	68.5
2. Which diseases/hazards can be transported to other areas via manure?	4.8	2.52	43.5
3. Which methods of manure treatment reduce the risks to animal and human health?	5.1	2.54	64.3
4. Right or wrong statements	4.1	1.69	67.8
Total score	18.1	5.92	58.4

%—Percentage. ^1^ Results are based on 101 participants. ^2^ SD—standard deviation. ^3^ Maximum possible score: for question 1, it was 6 points; for question 2, it was 11 points; for question 3, it was 8 points; and for question 4, it was 6 points. The maximum total score was 31.

**Table 4:** Determinants of farmers’ knowledge for safety manure management.

	Total Knowledge Score		
	β	95% CI	*p* *
Farming experience (years)	0.037	−0.07–0.10	0.69
Education level	0.192	0.06–3.47	0.043
Herd size (number of animals)	0.158	−0.17–2.48	0.087
Farming purpose	−0.497	−5.2–−2.36	<0.0005
R^2^	0.429	*p* **	<0.0005
Adjusted R^2^	0.402	F	16.131

The standard method for multiple linear regression was used. β—Standardised beta coefficients; CI—Confidence Interval; *p* *—significance of coefficients, *p* **—significance of the analysis.

**Table 5:** Comparison of knowledge scores of farmers with different attitudes and beliefs regarding safe manure management and farming practices.

Question about Attitudes and Beliefs	Statement	n	Median Score ^1^	*p*-Value ^2^	Effect ^3^ Size (*r*)
How important do you believe is the treatment of manure to reduce risks to animal or human health with each of the following methods before it can be used as a fertiliser?					
Storage in piles for at least 40 days with mixing for ventilation	Not important	26	16 (14, 21.5)	0.015	0.26
	Important	61	22 (19, 23)		
Storage in lagoons or tanks or tanks for 6 months	Not important	33	20 (16, 23)	0.016	0.29
	Important	35	22 (20, 24)		
Pasteurisation (70 °C for an hour)	Not important	21	21 (16, 23.5)	0.297	
	Important	37	22 (20, 23)		
Air-drying under the sun	Not important	14	16.5 (13.75, 23)	0.047	0.21
	Important	72	21 (19, 23)		
Composting	Not important	11	20 (14, 22)	0.049	0.23
	Important	62	21.5 (19, 23)		
Transformation into biogas	Not important	14	19.5 (15.5, 23.25)	0.097	
	Important	56	22 (20, 23)		
Addition of lime or other chemicals for sanitization purposes	Not important	18	21 (16, 23)	0.08	
	Important	51	22 (20, 23)		
How important do you consider the following actions for your farm?					
Vaccination program	Not important	17	16 (13,18.5)	0.001	0.33
	Important	80	21 (18,23)		
Mice control	Not important	11	11 (6, 14)	<0.0005	0.43
	Important	87	21 (18, 23)		
Increase in milk production	Not important	7	15 (12, 22)	0.042	0.22
	Important	80	21 (19, 23)		
Genetic improvement	Not important	7	12 (9, 16)	0.001	0.35
	Important	81	21 (18.5, 23)		
Production of feed	Not important	13	20 (14, 23.5)	0.378	
	Important	77	21 (18, 23)		
Animal welfare	Not important	6	13 (10.5, 17.25)	0.005	0.29
	Important	85	21 (17.5, 23)		
Manure management	Not important	12	16 (12.5, 20.75)	0.014	0.25
	Important	80	21 (18, 23)		

n—Number of total responses who chose the particular answer. ^1^ The score values are presented as median (interquartile score). ^2^ The *p*-value was obtained by the Mann–Whitney U Test. ^3^ The effect size refers to the Mann–Whitney U Test and is evaluated using Cohen [58] criteria of 0.1 = small effect, 0.3 = medium effect, 0.5 = large effect.

## Data Availability

Data will be available upon request by interested parties.

## Appendix A. The Questionnaire

I have read the information about the participation and agree to participate □

Part A: Demographics

1. Please state your age: ……………………
2. How many years have you been in the profession of bovine farmer? ……………………
3. What is the level of your education?

Primary (elementary)	
Gymnasium	
Lyceum	
University	
No	

4. In which district your farm is located? …………………………………………
5. Please note the animal population of your farm:

From 1 to 5 animals	
6–250	
251–500	
501–750	
751–1000	
More than 1000 animals	

6. What is the productive direction of your farm?

Dairy exclusively	
Fattening exclusively	
Mixed	
Local breed	

Part B: Assessment of the level of knowledge on the potential risks to human or animal health.

7. Please note which of the following do you believe help reducing the risk for the occurrence and transmission of diseases in animals or humans from improper manure management:

Vaccinations of animals for disease prevention	
Waiting for the manure to become digested	
Increase the milk yield with appropriate nutrition	
Genetic improvement of animals	
Production of feed on the farm	
Maintaining a high level of animal welfare	

8. Please note whether the following diseases can be transported to other areas via manure:

	YES	NO	I’m not sure
Mastitis (a disease that causes a decrease or cessation of lactation)			
Enteritis (a disease of the bowel, often with diarrhoea)			
Foot-and-mouth disease (disease with aphthae in the mouth and legs)			
Rabies			
Microbes that are resistant to antibiotics			
Ketosis (a disease that occurs during pregnancy or shortly after)			
Enteric parasites (mainly worms that live in the intestines or liver of animals)			
Pneumonia			
Mad cow disease			
Enterotoxaemia (diarrhoea with deaths in young animals)			
Tympany (Bloat=dilatation of the cattle stomach with gases)			

9. Below, some treatment methods for manure are listed. Please note in the appropriate column which of them you believe reduce the risks to animal and human health.

	Reduces	Does not reduce
The addition of lime		
Pasteurisation (70 °C for an hour)		
Air-drying		
Storage in lagoons or tanks or tanks		
Composting		
Storage in piles for at least 40 days with mixing for ventilation		
Transformation into biogas		
Separation of liquids from the solids		

10. Please note whether the following statements are Right or Wrong:

	R	W
- Manure that has been air dried for at least 90 days is safer than liquid manure.		
- Undigested manure can be placed on the land before or after sowing.		
- Uniform spreading of manure on land reduces risks.		
- The slope of the soil where liquid manure will be placed may affect safety because manure can be swept away elsewhere.		
- Raining does not affect the safety of manure dispersion on land.		
- After the manure is placed on land, at least week must elapse to harvest forage to be fed to cattle.		

Part C: Practices and approaches

11. How do you use/treat the manure produced in your farm?

Waste	
Fertilizer	
Energy Source	
Dangerous material	
Any other uses	

12. How often do you send the manure from your farm for final disposal?

Every Week	
Every month	
Every quarter	
More than three months	

13. How important do you believe is the treatment of manure to reduce risks to animal or human health with each of the following methods, before it can be used as a fertilizer?

	Not at All Important	Slightly Important	Important	Very Important	I Don’t Know
Storage in piles for at least 40 days with mixing for ventilation					
Storage in lagoons or tanks or tanks for 6 months.					
Pasteurisation (70 °C for an hour)					
Air-drying under the sun					
Composting					
Transformation into biogas					
Addition of lime or other chemicals for sanitization purposes					

14. To where do you dispatch the manure produced in your farm?

To be used directly as a fertiliser in the fields	
To a biogas plant	
To become fertilizer in a composting plant	
To be incinerated	
To be disposed of as a waste	

For another use. Please specify: …………………………………………………….

15. If you use manure as a fertilizer in the fields, this is in:

Liquid form	
Dried form	

16. How do you store the manure on your farm, until you send it to another place?

In a watertight tank	
In uncovered piles	
In covered piles	
I don’t store it at all	
In a dedicated area with cement floor	
In a place with earthen ground	
In any place that is convenient	

17. How many days does the manure stay stored at your farm before you send it to another place? …………
18. How important do you consider the following actions for your farm?

	Not Important	Slightly Important	Not Sure/No Opinion	Important	Very Important
Vaccination programme					
Mice control					
Increase in milk production					
Genetic improvement					
Production of feed					
Animal welfare					
Manure management					

Thank you very much for your participation!

## Appendix B. Additional Material

**Table A1:** Participants’ knowledge concerning safe manure management (Breakdown of the score to specific answers).

Question	Possible Answer	n	Correct Answers	%
Which of the following do you believe help reducing the risk for the occurrence and transmission of diseases in animals or humans from the improper management of the manure	Vaccinations of animals for disease prevention	101	61	60.4
	Waiting for the manure to become digested	101	55	54.5
	Increase the milk yield with appropriate nutrition	101	76	75.2
	Genetic improvement of animals	101	75	74.3
	Production of feed on the farm	101	81	80.2
	Maintaining a high level of animal welfare	101	58	57.4
Which of the following diseases/hazards can be transported to other areas via manure	Mastitis	93	56	60.2
	Enteritis	81	32	39.5
	Foot-and-mouth disease	84	41	48.8
	Rabies	81	31	38.3
	Microbes that are resistant to antibiotics	81	47	58
	Ketosis	80	56	70
	Intestinal parasites	82	45	54.9
	Pneumonia	80	48	60
	Mad cow disease	81	49	60.5
	Enterotoxaemia	80	37	46.3
	Tympany	84	53	63.1
Which of the following methods of manure treatment do you believe reduce the risks to animal and human health	The addition of lime	91	86	94.5
	Pasteurisation (70 °C for an hour)	78	64	82.1
	Air-drying	83	77	92.8
	Storage in lagoons or tanks or tanks	81	31	38.3
	Composting	77	63	81.8
	Storage in piles for at least 40 days with mixing for ventilation	84	67	79.8
	Transformation into biogas	84	74	88.1
	Separation of liquids from the solids	83	57	68.7
Right or Wrong Statements				
Manure that has been air dried for 90 days is safer than liquid manure.		97	89	91.8
Undigested manure can be placed on the land before or after sowing.		88	50	56.8
Uniform spreading of manure on land reduces risks		90	78	86.7
The slope of the soil where liquid manure will be placed may affect safety because manure can be swept away elsewhere.		90	70	77.8
Raining does not affect the safety of manure dispersion on land.		89	56	62.9
After the manure is placed on land, at least week must elapse to harvest forage to be fed to cattle.		91	69	75.8

n: Total responses for the specific possible answer; %: Percentage of the correct answers.

**Table A2:** Comparison of farmer groups with different demographic characteristics to the total knowledge score about safe manure management.

Participants’ Characteristics	Median Score ^1^	Effect Size ^2^ (*r*)	*p*-Value
Size of the farm n = 104			
Farms with equal or less than 250 bovines	19 (11, 22)	0.23	0.022 ^3^
Farms with more than 250 bovines	21 (19, 23)		
Farming purpose n = 103			
Pure Dairy or mixed with fattening	21 (18, 23)	0.49	<0.001
Pure fattening or local breeds	11 (7, 16)		
Participants’ educational level n = 104			
Farmers with lower education (elementary or gymnasium)	16 (8, 22)	0.22	0.028 ^3^
Farmers with higher education (lyceum or university)	20 (16, 23)		
Age n = 101			
Farmers up to 52 years old	20 (16, 23)	0.04	0.69 ^3^
Farmers older than 52 years old	21 (12, 23)		
Years in the occupation n = 99			
Farmers with experience equal or less than 25 years	19 (15, 23)	0.09	0.39 ^3^
Farmers with experience more than 25 years	21 (16, 23)		
Participants’ district n = 102			
Lefkosia	22 ^a^ (18, 23)		<0.001 ^4^
Ammochostos	20 ^a^ (14, 22.5)		
Larnaka	21 ^a^ (20, 23)		
Lemesos	21 ^a^ (19, 23)		
Paphos	12 ^b^ (7, 17)		

^1^ The score values are presented as median (interquartile score). ^2^ The effect size refers to the Mann–Whitney U-Test and is evaluated using Cohen (1988) criteria of 0.1 = small effect, 0.3 = medium effect, 0.5 = large effect. ^3^ The *p*-value was obtained by the Mann–Whitney U Test. ^4^ The *p*-value was obtained by the Kruskal–Wallis Test and associations between the groups with pairwise comparison. Different superscript letters denote statistical differences between groups.

## References

[1] EFSA (European Food Safety Authority) (2005). Opinion of the Scientific Panel on Biological Hazards (BIOHAZ) on the Biological Safety of Heat Treatment of Manure. EFSA J..

[2] Nag R., Russell L., Nolan S., Auer A., Markey B.K., Whyte P., O’Flaherty V., Bolton D., Fenton O., Richards K.G. (2022). Quantitative Microbial Risk Assessment Associated with Ready-to-Eat Salads Following the Application of Farmyard Manure and Slurry or Anaerobic Digestate to Arable Lands. Sci. Total Environ..

[3] Köninger J., Lugato E., Panagos P., Kochupillai M., Orgiazzi A., Briones M.J.I. (2021). Manure Management and Soil Biodiversity: Towards More Sustainable Food Systems in the EU. Agric. Syst..

[4] Stylianou M., Kaikiti K., Agapiou A. Sorption of Volatile Organic Compounds (VOCs) from Cattle Manure by Biochar. Proceedings of the 17th International Conference on Chemistry and the Environment.

[5] Rosen B.H., Croft R., Atwill E.R., Stehman S., Wade S. (2000). Waterborne Pathogens in Agricultural Watersheds.

[6] Wade S.E., Mohammed H.O., Schaaf S.L. (2000). Epidemiologic Study of Giardia Sp. Infection in Dairy Cattle in Southeastern New York State. Vet. Parasitol..

[7] Sobsey M.D., Khatib L.A., Hill V.R., Alocilja E., Pillai S. (2006). Pathogens in Animal Wastes and the Impacts of Waste Management Practices on Their Survival, Transport and Fate.

[8] Soller J.A., Schoen M.E., Bartrand T., Ravenscroft J.E., Ashbolt N.J. (2010). Estimated Human Health Risks from Exposure to Recreational Waters Impacted by Human and Non-Human Sources of Faecal Contamination. Water Res..

[9] USEPA (United States Environmental Protection Agency) Literature Review of Contaminants in Livestock and Poultry Manure and Implications for Water Quality. https://nepis.epa.gov/Exe/ZyPURL.cgi?Dockey=P100H2NI.txt.

[10] Manyi-Loh C.E., Mamphweli S.N., Meyer E.L., Makaka G., Simon M., Okoh A.I. (2016). An Overview of the Control of Bacterial Pathogens in Cattle Manure. Int. J. Environ. Res. Public Health.

[11] Geldreich E.E., Fox K.R., Goodrich J.A., Rice E.W., Clark R.M., Swerdlow D.L. (1992). Searching for a Water Supply Connection in the Cabool, Missouri Disease Outbreak of Escherichia Coli 0157:H7. Water Res..

[12] Solo-Gabriele H., Neumeister S. (1996). US Outbreaks of Cryptosporidiosis. J. Am. Water Works Assoc..

[13] Pell A.N. (1997). Manure and Microbes: Public and Animal Health Problem?. J. Dairy Sci..

[14] Khan S.J., Roser D.J., Davies C.M., Peters G.M., Stuetz R.M., Tucker R., Ashbolt N.J. (2008). Chemical Contaminants in Feedlot Wastes: Concentrations, Effects and Attenuation. Environ. Int..

[15] Pérez S., Barceló D., Aga D. (2008). Advances in the Analysis of Pharmaceuticals in the Aquatic Environment.

[16] Chee-Sanford J.C., Mackie R.I., Koike S., Krapac I.G., Lin Y.-F., Yannarell A.C., Maxwell S., Aminov R.I. (2009). Fate and Transport of Antibiotic Residues and Antibiotic Resistance Genes Following Land Application of Manure Waste. J. Environ. Qual..

[17] Spielmeyer A. (2018). Occurrence and Fate of Antibiotics in Manure during Manure Treatments: A Short Review. Sustain. Chem. Pharm..

[18] Muhammad J., Khan S., Su J.Q., Hesham A.E.L., Ditta A., Nawab J., Ali A. (2020). Antibiotics in Poultry Manure and Their Associated Health Issues: A Systematic Review. J. Soils Sediments.

[19] Hölzel C.S., Schwaiger K., Harms K., Küchenhoff H., Kunz A., Meyer K., Müller C., Bauer J. (2010). Sewage Sludge and Liquid Pig Manure as Possible Sources of Antibiotic Resistant Bacteria. Environ. Res..

[20] Udikovic-Kolic N., Wichmann F., Broderick N.A., Handelsman J. (2014). Bloom of Resident Antibiotic-Resistant Bacteria in Soil Following Manure Fertilization. Proc. Natl. Acad. Sci. USA.

[21] Oliver J.P., Gooch C.A., Lansing S., Schueler J., Hurst J.J., Sassoubre L., Crossette E.M., Aga D.S. (2020). Invited Review: Fate of Antibiotic Residues, Antibiotic-Resistant Bacteria, and Antibiotic Resistance Genes in US Dairy Manure Management Systems. J. Dairy Sci..

[22] Welsch-Pausch K., McLachlan M.S. (1998). Fate of Airborne Polychlorinated Dibenzo-p-Dioxins and Dibenzofurans in an Agricultural Ecosystem. Environ. Pollut..

[23] Koch F., Kowalczyk J., Wagner B., Klevenhusen F., Schenkel H., Lahrssen-Wiederholt M., Pieper R. (2021). Chemical Analysis of Materials Used in Pig Housing with Respect to the Safety of Products of Animal Origin. Animal.

[24] Gavinelli M.P. (2009). Persistance of Illegal Drugs in Bovine Manure. Doctoral Dissertation.

[25] Kjær J., Olsen P., Bach K., Barlebo H.C., Ingerslev F., Hansen M., Sørensen B.H. (2007). Leaching of Estrogenic Hormones from Manure-Treated Structured Soils. Environ. Sci. Technol..

[26] Andaluri G., Suri R.P.S., Kumar K. (2011). Occurrence of Estrogen Hormones in Biosolids, Animal Manure and Mushroom Compost. Environ. Monit. Assess..

[27] Combalbert S., Bellet V., Dabert P., Bernet N., Balaguer P., Hernandez-Raquet G. (2012). Fate of Steroid Hormones and Endocrine Activities in Swine Manure Disposal and Treatment Facilities. Water Res..

[28] Duerschner J., Bartelt-Hunt S., Eskridge K.M., Gilley J.E., Li X., Schmidt A.M., Snow D.D. (2020). Swine Slurry Characteristics as Affected by Selected Additives and Disinfectants. Environ. Pollut..

[29] Shao Z., Guo X., Qu Q., Kang K., Su Q., Wang C., Qiu L. (2021). Effects of Chlorine Disinfectants on the Microbial Community Structure and the Performance of Anaerobic Digestion of Swine Manure. Bioresour. Technol..

[30] Jahne M.A., Rogers S.W., Holsen T.M., Grimberg S.J., Ramler I.P. (2015). Emission and Dispersion of Bioaerosols from Dairy Manure Application Sites: Human Health Risk Assessment. Environ. Sci. Technol..

[31] Alegbeleye O.O., Sant’Ana A.S. (2020). Manure-Borne Pathogens as an Important Source of Water Contamination: An Update on the Dynamics of Pathogen Survival/Transport as Well as Practical Risk Mitigation Strategies. Int. J. Hyg. Environ. Health.

[32] Font-Palma C. (2019). Methods for the Treatment of Cattle Manure—A Review. C.

[33] Gollehon N.R., Caswell M., Ribaudo M., Kellogg R.L., Lander C., Letson D. (2001). Confined Animal Production and Manure Nutrients.

[34] Shortall O., Sutherland L., Ruralis A.R.-S., Kaler J. (2018). True Cowmen and Commercial Farmers: Exploring Vets’ and Dairy Farmers’ Contrasting Views of ’good Farming’in Relation to Biosecurity. Wiley Online Libr..

[35] Maye D., Chan K.W.R. (2020). On-Farm Biosecurity in Livestock Production: Farmer Behaviour, Cultural Identities and Practices of Care. Emerg. Top. Life Sci..

[36] Youssef D.M., Wieland B., Knight G.M., Lines J., Naylor N.R. (2021). The Effectiveness of Biosecurity Interventions in Reducing the Transmission of Bacteria from Livestock to Humans at the Farm Level: A Systematic Literature Review. Zoonoses Public Health.

[37] Liu Z., Wang X. (2020). Manure Treatment and Utilization in Production Systems. Animal Agriculture: Sustainability, Challenges and Innovations.

[38] Heinonen-Tanski H., Mohaibes M., Karinen P., Koivunen J. (2006). Methods to Reduce Pathogen Microorganisms in Manure. Livest. Sci..

[39] Collins E., Arogo J., Krometis L.A., Howes S. Evaluating the Effects of Pasteurization Temperature and Treatment Duration on Pathogen Inactivation in Separated Liquid Dairy Manure. Proceedings of the American Society of Agricultural and Biological Engineers Annual International Meeting.

[40] Nolan S., Thorn C.E., Ashekuzzaman S.M., Kavanagh I., Nag R., Bolton D., Cummins E., O’Flaherty V., Abram F., Richards K. (2020). Landspreading with Co-Digested Cattle Slurry, with or without Pasteurisation, as a Mitigation Strategy against Pathogen, Nutrient and Metal Contamination Associated with Untreated Slurry. Sci. Total Environ..

[41] Basnet B.B., Apan A.A., Raine S.R. (2002). Geographic Information System Based Manure Application Plan. J. Environ. Manage.

[42] Albihn A., Vinnerås B. (2007). Biosecurity and Arable Use of Manure and Biowaste—Treatment Alternatives. Livest. Sci..

[43] Bonetta S., Ferretti E., Bonetta S., Fezia G., Carraro E. (2011). Microbiological Contamination of Digested Products from Anaerobic Co-Digestion of Bovine Manure and Agricultural by-Products. Lett. Appl. Microbiol..

[44] Mc Carthy G., Lawlor P.G., Coffey L., Nolan T., Gutierrez M., Gardiner G.E. (2011). An Assessment of Pathogen Removal during Composting of the Separated Solid Fraction of Pig Manure. Bioresour. Technol..

[45] Loyon L. (2017). Overview of Manure Treatment in France. Waste Manag..

[46] Singh P., Gamal El-Din M., Ikehata K., Craik S.A., Bromley D. (2006). UV Inactivation of Bacteria in Raw and Pretreated Liquid Swine Manure. Environ. Technol..

[47] (2014). European Commission (DG Environment) Collection and Analysis of Data for the Control of Emissions from the Spreading of Manure Final Report. https://ec.europa.eu/environment/air/pdf/Final%20Report.pdf.

[48] de Corato U. (2020). Agricultural Waste Recycling in Horticultural Intensive Farming Systems by On-Farm Composting and Compost-Based Tea Application Improves Soil Quality and Plant Health: A Review under the Perspective of a Circular Economy. Sci. Total Environ..

[49] Polycarpou M. (2021). Personal communication.

[50] (2022). Department of Agriculture (Animal Production and Nutrition Branch) Overview of Cattle Breeding 2021; Lefkosia. http://www.moa.gov.cy/moa/da/da.nsf/All/AAD5881263760756C22587A600338D12?OpenDocument.

[51] Valeeva N.I., van Asseldonk M.A.P.M., Backus G.B.C. (2011). Perceived Risk and Strategy Efficacy as Motivators of Risk Management Strategy Adoption to Prevent Animal Diseases in Pig Farming. Prev. Vet. Med..

[52] Tierney M., Gallagher A.M., Giotis E.S., Pentieva K. (2017). An Online Survey on Consumer Knowledge and Understanding of Added Sugars. Nutrients.

[53] Boivin X., Marcantognini L., Boulesteix P., Godet J., Brulé A., Veissier I. (2007). Attitudes of Farmers towards Limousin Cattle and Their Handling. Anim. Welf. J..

[54] Jansen J., van den Borne B.H.P., Renes R.J., van Schaik G., Lam T.J.G.M., Leeuwis C. (2009). Explaining Mastitis Incidence in Dutch Dairy Farming: The Influence of Farmers’ Attitudes and Behaviour. Prev. Vet. Med..

[55] (2019). IBM SPSS Statistics for Windows 2019, v.27.0.

[56] Casal J., de Manuel A., Mateu E., Martín M. (2007). Biosecurity Measures on Swine Farms in Spain: Perceptions by Farmers and Their Relationship to Current on-Farm Measures. Prev. Vet. Med..

[57] Benjamin L.A., Fosgate G.T., Ward M.P., Roussel A.J., Feagin R.A., Schwartz A.L. (2010). Attitudes towards Biosecurity Practices Relevant to Johne’s Disease Control on Beef Cattle Farms. Prev. Vet. Med..

[58] Cohen J. (1988). Statistical Power Analysis for the Behavioral Sciences.

[59] Ström G., Albihn A., Jinnerot T., Boqvist S., Andersson-Djurfeldt A., Sokerya S., Osbjer K., San S., Davun H., Magnusson U. (2018). Manure Management and Public Health: Sanitary and Socio-Economic Aspects among Urban Livestock-Keepers in Cambodia. Sci. Total Environ..

[60] Šūmane S., Kunda I., Knickel K., Strauss A., Tisenkopfs T., des los Rios I., Rivera M., Chebach T., Ashkenazy A. (2018). Local and Farmers’ Knowledge Matters! How Integrating Informal and Formal Knowledge Enhances Sustainable and Resilient Agriculture. J. Rural. Stud..

[61] Eltayb A., Barakat S., Marrone G., Shaddad S., Stålsby Lundborg C. (2012). Antibiotic Use and Resistance in Animal Farming: A Quantitative and Qualitative Study on Knowledge and Practices among Farmers in Khartoum, Sudan. Zoonoses Public Health.

[62] Calistri P., D’Albenzio S., Falconi A., Pediconi O., Johnson J., Vincent A. (2021). 2021 EFSA/IZSAM Animal Health Crisis Preparedness Exercise with Mediterranean Countries. EFSA Support. Publ..

[63] Sahlström L., Bagge E., Emmoth E., Holmqvist A., Danielsson-Tham M.L., Albihn A. (2008). A Laboratory Study of Survival of Selected Microorganisms after Heat Treatment of Biowaste Used in Biogas Plants. Bioresour. Technol..

[64] Liu X., Lendormi T., Lanoisellé J.L. (2021). Conventional and Innovative Hygienization of Feedstock for Biogas Production: Resistance of Indicator Bacteria to Thermal Pasteurization, Pulsed Electric Field Treatment, and Anaerobic Digestion. Energies.

[65] Meyer D., Price P.L., Rossow H.A., Silva-del-Rio N., Karle B.M., Robinson P.H., DePeters E.J., Fadel J.G. (2011). Survey of Dairy Housing and Manure Management Practices in California. J. Dairy Sci..

[66] Nandiyanto A.B.D., Ragadhita R., Maulana A.C., Abdullah A.G. (2018). Feasibility Study on the Production of Biogas in Dairy Farming. IOP Conf. Ser. Mater. Sci. Eng..

[67] Gebrezgabher S.A., Meuwissen M.P.M., Prins B.A.M., Lansink A.G.J.M.O. (2021). Economic Analysis of Anaerobic Digestion—A Case of Green Power Biogas Plant in The Netherlands. NJAS Wagening. J. Life Sci..

[68] Pochwatka P., Kowalczyk-Juśko A., Sołowiej P., Wawrzyniak A., Dach J. (2020). Biogas Plant Exploitation in a Middle-Sized Dairy Farm in Poland: Energetic and Economic Aspects. Energies.

[69] Coats E.R., Gregg M., Crawford R.L. (2011). Effect of Organic Loading and Retention Time on Dairy Manure Fermentation. Bioresour. Technol..

[70] Water Pollution Control Law ΚΔΠ 433/2006, The Water Pollution Control General Conditions for Disposing of Waste from Cattle Farming Units, Decree 2006, Cyprus Government Gazette, 4148, *annex III(1)*, 3701–3714. https://www.mof.gov.cy/mof/gpo/gazette.nsf/All/58C81A479097F4B9C2258728003A2228/$file/4148%2017.11.2006%20Parartima%203o%20Meros%20I.pdf?OpenElement.

[71] Wang W., Jin J., He R., Gong H. (2017). Gender Differences in Pesticide Use Knowledge, Risk Awareness and Practices in Chinese Farmers. Sci. Total Environ..

[72] Singh B.B., Kaur R., Gill G.S., Gill J.P.S., Soni R.K., Aulakh R.S. (2019). Knowledge, Attitude and Practices Relating to Zoonotic Diseases among Livestock Farmers in Punjab, India. Acta Trop..

[73] Hundal J.S., Sodhi S.S., Gupta A., Singh J., Chahal U.S. (2016). Awareness, Knowledge, and Risks of Zoonotic Diseases among Livestock Farmers in Punjab. Vet. World.

[74] Saha D., Hoque Akand A., Hai A. (2010). Livestock Farmers’ Knowledge about Rearing Practices in Ganderbal District of Jammu & Kashmir. Indian Res. J. Ext. Edu..

[75] Musallam I.I., Abo-Shehada M.N., Guitian J. (2015). Knowledge, Attitudes, and Practices Associated with Brucellosis in Livestock Owners in Jordan. Am. J. Trop. Med. Hyg..

[76] Holt H.R., Eltholth M.M., Hegazy Y.M., El-Tras W.F., Tayel A.A., Guitian J. (2011). *Brucella* Spp. Infection in Large Ruminants in an Endemic Area of Egypt: Cross-Sectional Study Investigating Seroprevalence, Risk Factors and Livestock Owner’s Knowledge, Attitudes and Practices (KAPs). BMC Public Health.

[77] Gozdzielewska L., King C., Flowers P., Mellor D., Dunlop P., Price L. (2020). Scoping Review of Approaches for Improving Antimicrobial Stewardship in Livestock Farmers and Veterinarians. Prev. Vet. Med..

[78] Nöremark M., Lindberg A., Vågsholm I., Sternberg Lewerin S. (2009). Disease Awareness, Information Retrieval and Change in Biosecurity Routines among Pig Farmers in Association with the First PRRS Outbreak in Sweden. Prev. Vet. Med..

